# Fisher-Level Decision Making to Participate in Fisheries Improvement Projects (FIPs) for Yellowfin Tuna in the Philippines

**DOI:** 10.1371/journal.pone.0163537

**Published:** 2016-10-12

**Authors:** Frazen Tolentino-Zondervan, Paul Berentsen, Simon R. Bush, Larry Digal, Alfons Oude Lansink

**Affiliations:** 1 Business Economics Group, Wageningen University, Wageningen, The Netherlands; 2 Environmental Policy Group, Wageningen University, Wageningen, The Netherlands; 3 School of Management, University of the Philippines in Mindanao, Davao del Sur, Philippines, Tuna Value Chain Project, Commission on Higher Education, Davao City, Philippines; James Cook University, AUSTRALIA

## Abstract

This study identifies the capabilities needed by small-scale fishers to participate in Fishery Improvement Projects (FIPs) for yellowfin tuna in the Philippines. The current literature provides little empirical evidence on how different models, or types of FIPs, influence the participation of fishers in their programs and the degree which FIPs are able to foster improvements in fishing practices. To address this literature gap, two different FIPs are empirically analysed, each with different approaches for fostering improvement. The first is the non-governmental organisation-led Partnership Programme Towards Sustainable Tuna, which adopts a bottom-up or development oriented FIP model. The second is the private-led Artesmar FIP, which adopts a top-down or market-oriented FIP approach. The data were obtained from 350 fishers surveyed and were analysed using two separate models run in succession, taking into consideration full, partial, and non-participation in the two FIPs. The results demonstrate that different types of capabilities are required in order to participate in different FIP models. Individual firm capabilities are more important for fishers participation in market-oriented FIPs, which use direct economic incentives to encourage improvements in fisher practices. Collective capabilities are more important for fishers to participate in development-oriented FIPs, which drive improvement by supporting fishers, fisher associations, and governments to move towards market requirements.

## Introduction

The sustainability of fisheries is driven in large part by the alignment of fisher practices with management goals [1, 2]. State regulations, such as restrictions on fishing gears, harvest control rules, and access restrictions have traditionally been applied to change fisher behavior. However, the perceived weakness of these state regulations, or total absence in many developing country fisheries (DCFs), has led to the emergence of private incentive mechanisms, which are designed to improve compliance with existing rules and management approaches [3–5]. The design and objectives of these private incentive mechanisms differ, but most commonly involve incentivising changes in fishing practices through value chain based arrangements such as industrial coalitions, improvement projects, and eco-certification (e.g. [6]).

The most dominant private incentive mechanisms for sustainable fisheries is the Marine Stewardship Council (MSC) standards, against which fisheries practices, the health of stocks and habitats, and the capacity of management to deliver sustainable outcomes are measured and certified [7]. However, one of the main criticisms of the MSC is its limited capacity to adequately include DCFs. As of 2015, only 8% MSC-certified fisheries are from developing countries [8]. This limited inclusion is attributed to the high cost of certification, the lack of data on fish stocks available for assessment, and the inadequate or absence of effective governance and regulatory systems [9–12]. Recognising the difficulties of DCFs to move towards certification, a range of Non-Government Organisations (NGOs) and private consultancy firms have developed Fishery Improvement Projects (FIPs), a step-wise methodology for improving fisheries practices and management that originally started in developed world contexts but is also focused on DCFs [13–15].

FIPs utilize the market incentives in seafood value chains to stimulate sustainability improvements, which may or may not lead to MSC certification [15]. For example, retailers and food companies can push fishers towards improvements by directly funding a FIP or purchasing products (with or without a premium) from a fishery in a FIP [16]. The six stages FIP model proposed by the Sustainable Fisheries Partnership is as follows: 1. the identification of improvement goals and engagement of corporate partners; 2. agreement on work plans for improvement between fishers and participating partners; 3. engagement of regulators by FIP partners to improve regulation and market partners to adopt better product specification and procurement policies; 4. measurement of improvements to policy and practice; 5. key scientific indicators demonstrating a positive trend towards management goals; and 6. (optional) certification against the MSC standards [17]. While differing in substance, other FIP models demonstrate a similar logic (see [18]) and are predicated on facilitating access to high end markets under the *notional* condition [15] that the fishery is working towards improvement.

Despite convergence around the type of steps required, the mix of organisations involved, the kinds of fishers targeted, and the extent of institutional support provided in FIPs differ considerably. This is especially the case in the estimated 130-plus developing country FIPs [15]. Based on a recent attempt to create a general classification of FIP implementation [19] we define two general categories: ‘bottom-up’ development-oriented FIPs, often led by NGOs stimulating general improvements to government support and regulation; and ‘top-down’ market-oriented FIPs, led by firms focused on direct economic benefits for fishers in return for strict compliance [19]. It is assumed that these FIP models have consequences for the way fishers are included in FIP programs, especially in terms of the decision of fishers to change their practices in accordance with improvement criteria. Yet, there is little empirical evidence to verify this [14, 15].

We argue that the decisions made by fishers to participate in a FIP depends on the type of capabilities they have and whether these capabilities match with the requirements for participation. These capabilities refer to the specific skills, practices, and forms of social organisations [20, 21]. This study classifies these capabilities into individual capabilities at personal level, individual capabilities at firm-level, and collective capabilities at the fishery or community level. By identifying these capabilities we build a clearer understanding of the specific factors that influence fisher decisions to comply with requirements that seek to improve fishing practices.

The objective of this paper is to determine which decision making factors are important for small scale Filipino tuna fishers’ decisions to participate in two FIPs for yellowfin tuna (*Thunnus albacares*) in the Philippines. The first is the market-oriented Artesmar FIP, which is run by the private-company Meliomar and the consultancy firm BlueYou (Switzerland). This FIP sets a high sustainability requirement and provides economic incentives to encourage fishers participation. The second is the development-oriented Partnership Programme Towards Sustainable Tuna (PPTST) FIP, which is run by the World Wildlife Fund for Nature (WWF) Philippines, and seeks improved local governance of tuna fisheries to meet global value chain requirements. Both FIPs are focused on yellowfin tuna because of its market value and importance to the local economy, the scalability of these FIPs to other sites in the Philippines and beyond targeting yellowfin tuna, and because yellowfin is a species subject to overfishing in recent years [22–24].

To understand the factors that influence fisher’s decisions to participate in FIPs, we employ a two stage framework including two models which are run separately. The following section describes both FIP models and the theoretical basis of capabilities and decision making. This is followed by an outline of the empirical data collection and of the probit and ordered probit models used for the two stage modelling. We then provide a justification for the variables adopted to explain fisher participation in FIPs. The paper concludes with a discussion of the key variables that are important for participation in different models of FIPs and recommendations for enhancing fisher participation in FIPs.

## Participation in Different FIP Models

### Comparative FIP models

In line with defining the different FIP models, the California Environmental Associates (CEA) [19] created four archetypes based on combination of two characteristic dimensions. The first dimension focuses on the structure of FIPs, ranging from ‘basic’ to ‘comprehensive’. Basic FIPs are characterised as a simple, low-cost model which provides small incremental improvements through time, while comprehensive FIPs are considered those that are resource intensive model and aimed at achieving MSC certification. The second dimension focuses on supply chain engagement along a spectrum of bottom-up vs. top-down. Bottom-up FIPs are those that develop improvements first and only later attempt to access high end markets and major buyers who have made sustainability commitments [19]. In contrast, top-down FIPs are those that start with the demand of major buyers and retailers to put pressure on fisheries to engage in sustainability in exchange for market access. The four possible archetypes from these two dimensions allow us to classify Artesmar as a top-down comprehensive FIP and PPTST as a bottom-up comprehensive FIP.

**Fig 1:**
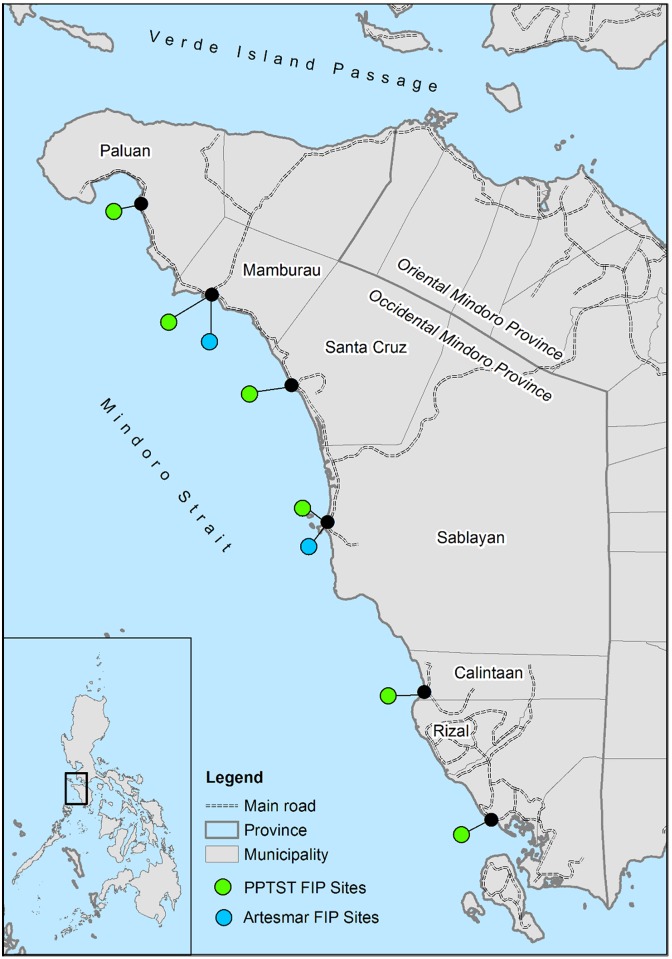
The Occidental Mindoro fishery map.

Beginning in 2013, the goal of Artesmar is to provide market recognition and incentives for improved business and fishing practices of small scale fishers in the Philippines. Artesmar works in different regions of the Philippines, trading yellowfin from Occidental Mindoro, Albay, Quezon and Infanta, Antique, and Eastern Samar, Palawan, Batangas, Subic, Negros Occidental, and Zamboanga. Our research focused on Sablayan and Mamburao municipalities in Occidental Mindoro (see Fig 1) because the export chain for yellowfin tuna has been established since 2010 and full participation in the FIP is observable. Both of these municipalities are characterised by a higher concentration of fishers with larger tuna boats and higher tuna landings than the other municipalities of Occidental Mindoro. The two municipalities also have good access to tuna processing plants due to improved roads, proximity to landing sites for easier tuna transfer, and the availability of transportation and major services such as communication and electricity which facilitates improved business transactions.

The Artesmar FIP supports fishers to be compliant with legal catch documentation requirements, as well as enhancing fishery data, and fishery co-management; all of which are necessary to prepare the fishery for MSC certification. Artesmar also sets chain of custody requirements, including strict quality standards for tuna, and traceability requirements such as vessel registration and full catch documentation to verify the absence of Illegal Unreported Unregulated (IUU) fishing. Fishers participating in the Artesmar FIP are more likely to receive higher prices for their fish compared to fishers who do not participate because they are trained in how to handle their catch in such a way that improves the quality of the meat. They also have more certainty of having a buyer and receiving timely payment for the fish they land. These benefits of participation in the Artesmar FIP lower the risk of having a highly variable income, but are offset by the investment required for participation including time and effort allocated to training, additional effort and investment to satisfy traceability requirements and upgrading facilities to meet fish safety and quality requirements. The cost of training is particularly high because of the structure of boat ownership, with fishers owning several boats having to extend new equipment, practices, and knowledge to their multiple boats and boat captains.

The PPTST FIP is based on a public-private partnership established in 2011 to develop sustainable practices of yellowfin tuna fishers in Lagonoy and in Occidental Mindoro. Although funded by WWF Germany and European retailers such as Seafresh (Netherlands), Bell Seafood (Germany), and Coop (Switzerland), neither higher landing prices nor market access is currently used as an incentive to participate in the FIP. Instead the PPTST FIP can be classified as a bottom-up comprehensive FIP, with day-to-day management of the FIP carried out by the WWF-Philippines and the municipal governments to improve the wider conditions of legal compliance and fisher safety in the fishery. Implementation of the PPTST FIP by WWF and the municipal governments focuses primarily on the organisation of fishers in associations, before engaging them in a consultative decision making process in order to comply with chain requirements similar to the Artesmar FIP (e.g. [25, 26]).

The PPTST FIP targets a very wide range of fishers across the six municipalities of Sablayan, Mamburao, Paluan, Sta. Cruz, Calintaan, and Rizal, Occidental Mindoro (see Fig 1). Fishers in Sablayan and Mamburao are large scale and have adequate capital to meet requirements set by buyers in Metro Manila. Fishers in the other four municipalities have smaller scale operations, target species other than yellowfin tuna, and have other forms of livelihoods such as rice farming, carpentry, and grocery stores. Unlike the Artesmar FIP which sets strict requirements for inclusion in the FIP, fishers can partially participate in the PPTST FIP by attending trainings related to fishery governance, without delivering tuna to the chain. Full participation requires the preparation of catch documents and providing export quality tuna based on their attendance at training sessions. In general, institutional support such as training, subsidies, and in-kind help are recognised as important indirect incentives for the improvement of fisher practices (see [6]). Contrary to the trainings in the Artesmar FIP, the trainings in the PPTST FIP are organized and funded completely by external actors such as the WWF and the municipalities. The PPTST trainings are not only limited to complying with tuna quality and traceability requirements, but also extend to improving the governance of the fishery, such as putting in place anti-IUU measures, and supporting the development of alternative livelihoods (such as ecotourism). Moreover these trainings reach different types of fishers ranging from small- to large-scale, including those fishers in remote areas of the municipalities. The trainings, subsidies, and in-kind help are considered indirect incentives of the bottom up comprehensive FIPs because they are not directly associated with the market incentives of increased market access or a price premiums [6].

### Fishers’ participation decision framework

**Fig 2:**
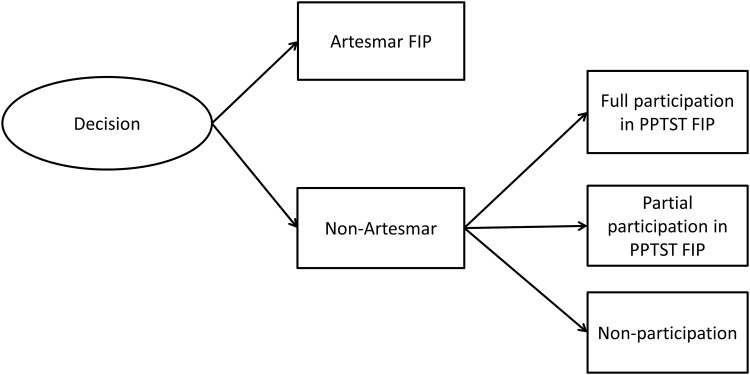
The decision model for fisher participation.

This study analyses fishers’ decisions to participate in the two FIPs (Fig 2). The first stage decision concerns the choice to opt for participation in the Artesmar FIP or not. In principle, the Artesmar FIP delivers higher returns and lowers the risk of fishers in terms of fluctuation in fish prices. However, higher investments for participation are required. The basic assumption here is that fishers who can fulfil the requirements of Artesmar FIP will choose this alternative because this option is expected to lead to a higher utility. As explained by the Utility Maximisation (UM) framework, a rational individual will maximise his/her income and will minimise risks [27–29]. The second assumption is that participation in the Artesmar FIP, though preferred by fishers, is not feasible for many fishers due to their lack of capabilities to comply with the requirements of the Artesmar FIP. Fishers that find themselves unable to participate in the Artesmar FIP might then opt for participation in the PPTST FIP as a second best option. Participation of a fisher in the PPTST FIP can be partial or full, depending on the fulfilment of requirements. As an extension of the utility framework, the third assumption is that the perceived social benefits of community membership (see [30–32]), adds to the utility of fishers when joining the PPTST FIP. The final option is that a fisher does not participate in any FIP.

Testing the participation choices requires identifying significant variables that affect income and risks of fishers. The literature on decision making has extensively described the characteristics of decision makers that affect their level of income and risk (e.g. [33–36]). For this study, we divide these characteristics into four groups: individual capabilities at personal level, individual capabilities at firm-level, collective capabilities, and individual risk attitude and socio-demographic characteristics. Table 1 shows this grouping of characteristics, which are also used as the explanatory variables used to analyse decisions for FIP participation in the empirical model explained below.

**Table 1:** The explanatory variables for fishers’ participation in Artesmar and PPTST FIPs.

Characteristics of decision makers	Definition of variables in Stata	References
FIP	1 if a fisher belongs to non-Artesmar, 0 if a fisher belongs to Artesmar	
Stages	1 if fisher belongs to non-participation, 2 if partial participation in PPTST FIP, and 3 if full participation in PPTST FIP	
*1*. *Individual personal capabilities*		
Fishing years	Fishing experiences (in years)	[34, 39, 47]
Education	Educational attainment of fisher (1 if fisher reaches high school, 0 otherwise)	[34, 39, 47]
*2*. *Individual firm capabilities*		
Initial investment	Initial investment for tuna fishing boat (in ‘000,000 Philippine Pesos)	[34, 76]
Boat ownership	1 if tuna fishing boat owner, 0 if non-boat owner	[6]
Boat capacity	Tuna capacity of boats (in ‘000 kg)	[6]
Fishing trips	Number of tuna fishing trips in a month	[42]
Type of fishing employment	1 if fisher is fishing tuna year round, 0 otherwise	[42]
Operating distance	Tuna fishing operating distance (in km)	[42]
Fishing days	Number of tuna fishing days	[42]
*3*. *Collective capabilities*		
Membership to an association	1 if fisher has membership to a fisher association, 0 otherwise	Interviews, [6]
Trainings and subsidies	1 if a fisher receives trainings and subsidies from government, 0 otherwise	Interviews, [6]
Financing operation	1 if fisher personally finances his fishing operation, 0 if finances by *Casas*	Interviews, [6, 43, 44, 50]
*4*. *Individual risk attitude and socio demographic*		
Risk Attitude	1 if fisher sells in quality method, 0 if straight method	[34, 35, 46]
Age	Individual fisher’s age (in years)	[34, 47]
Number of family members	Number of immediate family members	[34]
Other source of income	1 if fisher has other sources of income, 0 otherwise	[48, 49]

Capabilities are defined as the specific skills, practices, and forms of social organisations required to deliver certain tasks in pursuit of long-term goals [20, 21]. The level of capabilities of individuals or firms influences the amount of risk they can handle and the income they can generate [37]. For instance, a fisher that is more capable of adopting a certain fishing technology will find the risk of adoption lower than a fisher who still needs to build his/her capability to adopt the fishing technology. Moreover, the adoption of a fishing technology by a more capable fisher increases the likelihood of gaining a higher income [34, 35, 38].

The capabilities are expressed at the individual, firm and collective levels. As shown in Table 1, individual capabilities of the fishers correspond to what others have termed human capital, including the experiences and education of an individual, and can be used as a proxy for the competences and skills either inherent or acquired by that individual [39]. Firm capabilities refer to the collection of competences of the organisation to which the individual belongs [39–41] and reflect the use of material resources in order to comply with transaction requirements [6]. In this study boats are considered as the unit of a firm operated by individuals. The material resources that are translated to individual firm capabilities include initial investments, boat ownership, boat capacity, type of fishing employment, number of fishing trips, operating distance, and fishing days [6]. These last three factors represent the input variables used by fishers, including gasoline/fuel, ice, and labor costs [42]. Collective capabilities correspond to what others refer to as social capital, including shared resources acquired through external relationships and networks that improve personal- and firm-level capabilities [39]. In practical terms this extends to membership to a fisher association, institutional support such as trainings and subsidies, and financing business operations through loans either from an association or from *Casas*–local elites or patrons who control trade and provide credit for fishing, as well as for household needs such as education and health (see [43–45]). Based on compliance with market requirements the Artesmar FIP demands a higher level of capabilities for participation. This study hypothesizes that individual capabilities at firm-level will increase the likelihood of fisher participation to the Artesmar FIP because these variables reflect the higher capabilities of fishers.

The individual risk attitude and socio-demographic variables influence the required income and acceptable risk for the decision maker [34, 35]. This group of variables includes risk attitude, age, number of family members, and other sources of income (Table 1). The risk attitude reflects the extent to which decision makers value risks [46]. A value of 1 in the variable ‘risk attitude’ (Table 1) corresponds to the risk fishers take on by having a preference for what is termed locally as ‘quality buying’—a method that requires quality inspection of tuna and assigns higher price for export quality tuna while lower price for rejected quality tuna. In this study, fishers with lower risk aversion (i.e. risk takers) will more likely choose the alternative that will give a higher expected income [33, 34, 38]. This study assumes that socio-demographic variables may result in two opposite effects, which can influence the significance of these variables in the model. For instance, the age of the fisher affects the time horizon with which an investment can be recovered [34, 47]. A higher age is therefore expected to decrease the likelihood for participation in the Artesmar FIP. At the same time, a higher age may also mean fishers have more fishing experience to improve their compliance with market requirements and could therefore increase the likelihood of participation in the Artesmar FIP [34]. Similarly a higher number of family members may reduce the risk of a fisher or firm by increasing the availability of labour, thus increase the likelihood of participation in the Artesmar FIP [34]. However, the number of family members also places greater pressure to remain economically viable and food secure, which can lead to a lower probability of participation in the Artesmar FIP. Additional sources of income may reduce this risk by providing alternative sources of finance to firms, which in turn may increase the funds available and the likelihood of participation in the Artesmar FIP [48]. At the same time, other sources of income are also assumed to increase risk due to the allocation of time and resources to other activities, thus decreasing the likelihood of participation in the Artesmar FIP [49].

In the second-stage decision, collective capabilities such as membership to an association, and subsidies from the government enable fishers to comply with the requirements of the development-oriented FIP, while the financial dependency on *Casas* is either improving or limiting the participation of fishers. These collective capabilities reduce the exposure of fishers to risks by acquiring parts of investments to external actors and developing fishers’ capabilities through bottom-up comprehensive FIPs, and thus improve their income. We assume that membership to a fisher association strengthens the social relationships among fishers, enables fishers to improve the management of their fisheries, and allows access to the external resources that help fishers to improve their fishing activities in order to comply with FIP requirements [6]. Fishers, therefore, will perceive higher utility in joining a bottom-up comprehensive FIP given their focus on establishing fisher associations. Finally, we assume that the financial dependency of fishers to *Casas* can motivate fishers to participate in these FIPs because of (1) fishers feelings of indebtedness to *Casas* and/or (2) the (mis)trust of *Casas* that prices are correlated with the quality of fish. At the same time, the financial dependency of fishers to *Casas* may limit their freedom in joining a FIP in which they are not involved, because *Casas* may lose control over fishers which is based on a mix of familial and debt-tied relations [43, 44, 50].

The non-participation of fishers in all FIPs may not only be attributable to a lack of capabilities only, but also to a higher perceived risk of participating in a FIP. Field results indicate that the risk perception of fishers is mainly influenced by four factors: 1) the existing tied-credit relation of fishers to *Casas* that results to lower fish price, while leaving no alternative higher markets to fishers for selling their fish; and by 2) the lack of trusts of fishers in those assessing quality at landing sites [50]; (3) the increasing temperature that results to low tuna quality; and (4) the decreasing tuna catches in the recent years that causes fluctuation in income [51].

Based on the assumptions outlined here, the rest of the paper addresses two key questions. First, which variables are important for fishers participation in the Artesmar and PPTST FIPs? Second, based on these variables, to what extent do top down and bottom up comprehensive FIPs facilitate participation of fishers in their programs?

## Materials and Methods

### Data Collection

Individual fisher data were obtained from a survey covering the six municipalities of Occidental Mindoro. The Wageningen School of Social Sciences (WASS) at Wageningen University assessed and approved the research proposal in 2013 before fieldwork commenced in 2014. Verbal consent was sought from fishers by first explaining to them the objective of this study, the kind of information this study requires, and the use of the information they will provide. The interviews commenced after verbal consent was given and recorded by including the name of the fishers in the survey form. Written consent was not obtained due to the illiteracy of most of the fishers and the sensitivity of some fishers in terms of signing any form of what they perceive to be formal documentation containing their names.

The total fisher population in Occidental Mindoro is 3584 fishers, according to the database of WWF Philippines as of June 2014. Following Field [52], a total of 350 randomly selected surveys were carried out with the target of 10% of the population across spatially dispersed areas per municipality (see Table 2). The number of complete responses was 316, which can be divided into four groups according to their participation in the Artesmar FIP (6.3%), full participation in the PPTST FIP (17.4%), partial participation in the PPTST FIP (34.2%), and non-participation (42.1%) (see Table 3). The number of Artesmar FIP fishers is partly limited since the Artesmar FIP started two years later than the PPTST FIP and only covered two out of six FIP municipalities in Occidental Mindoro.

**Table 2:** The distribution of samples in each municipality.

Municipality	Total population**	Number of samples*
Sablayan	1656	165
Mamburao	850	85
Paluan	113	12
Sta. Cruz	350	25
Calintaan	315	33
Rizal	300	30
Total	3584	350

*Total number of surveys carried out is 350. However, the number is reduced to 316 due to incomplete and non-responses of some fishers.

**Source: WWF Philippines, June 2014

**Table 3:** Descriptive statistics of variables based on four groups of fishers participation in FIP.

Variables		Artesmar FIP (*n = 20) 6*.*3%*	Full PPTST FIP (*n = 55) 17*.*4%*	Partial PPTST FIP (*n = 108) 34*.*2%*	No Participation (*n = 133) 42*.*1%*
*Scale variables*					
Fishing years		23.95 (2.7410)	21.50 (1.6487)	19.03 (1.09)	18.81 (0.9835)
Initial investments		3.79 (2.2136)	1.98 (0.3295)	0.61(0.1090)	0.74 (0.1423)
Boat capacity		1.74 (0.3172)	1.25 (0.1280)	0.90 (0.1575)	0.90 (0.0948)
Number of fishing trips		3.9 (1.0709)	3.60 (0.3349)	8.96 (0.8252)	7.67 (0.7033)
Operating distance		46.25 (9.9331)	50.27 (5.3346)	35.10 (2.8944)	35.54 (2.6733)
Number of fish days		6.6 (0.6257)	6.05 (0.3571)	4.15 (0.2737)	4.69 (0.2309)
Age		44.75 (2.8533)	42.45 (1.3530)	41.81 (1.1230)	40.53 (0.9858)
Number of family members		6.25 (0.5020)	4.96 (0.2854)	5.35 (0.2245)	5.16 (0.1801)
*Nominal variables*		*Frequency count (%)*	*Frequency count (%)*	*Frequency count (%)*	*Frequency count (%)*
Education	Reach Hs (1)	10 (50)	55 (50.9)	57 (42.9)	30 (54.5)
	Do not reach Hs (0)	10 (50)	53 (49.1)	76 (57.1)	25 (45.5)
Membership to an association	Yes (1)	4 (20)	67 (62.0)	12 (9.0)	44 (80.0)
	No (0)	16 (80)	41 (38.0)	121 (90.10)	11 (20.0)
Trainings and subsidies	Yes (1)	3 (0.15)	55 (50.9)	17 (12.8)	22 (40.0)
	No (0)	17 (0.85)	53 (49.1)	116 (87.2)	33 (60.0)
Boat ownership	Boat owner (1)	8 (40)	78 (72.2)	71 (53.4)	42 (76.4)
	Not owner (0)	12 (60)	30 (27.8)	62 (46.6)	13 (23.6)
Financing operation	Personal (1)	8 (0.4)	61 (56.5)	70 (52.6)	18 (32.7)
	Casa (0)	12 (0.6)	47 (43.5)	63 (47.4)	37 (67.3)
Season	Full-time (1)	8 (40)	34 (31.5)	51 (38.3)	46 (83.6)
	Part-time (0)	12 (60)	74 (68.5)	82 (61.7)	9 (16.4)
Risk attitude	Quality (1) buying	9 (0.45)	13 (12.0)	11 (8.3)	18 (32.7)
	Straight (0) buying	11 (0.55)	95 (88.0)	122 (91.7)	37 (67.3)
Source of income	Yes (1)	10 (50)	78 (72.2)	67 (50.4)	25 (45.5)
	No (0)	10 (50)	30 (27.8)	66 (49.6)	30 (54.5)

Table 3 also shows descriptive statistics of the independent variables that were used to explain fisher participation. In general, the scale variables such as fishing years, initial investments, boat capacity, operating distance, number of fish days, age, and number of family members show an increasing trend with increasing levels of participation, while the number of fishing trips shows a decreasing trend. The distribution of fishers is also shown for each nominal variable. The education level of fishers is evenly distributed across four fisher participation options. The membership to a fisher association and the boat ownership of fishers increase from non-participation to full participation in the PPTST FIP. Artesmar FIP has the highest fraction of risk taking fishers. Also the percentage of fishers that prefers quality buying increases with the degree of participation in the PPTST FIP.

### The empirical model

In modelling fishers’ decision making to participate in FIPs, fishers decisions are structured in the two stages as explained above (Fig 2). The initial explanatory variables to explain fisher’s decision making are outlined in Table 1 and are coded in Stata 13, the statistical program used in this paper.

We employed a two-stage modelling approach, similar to the concept of Heckman’s two-stage sample selection model [53–56], because it enables us to assess fisher’s participation in FIPs in two stages [57, 58]. The first stage is a sample selection equation that uses a probit model. In this study, the sample selection deals with fishers options to choose between Artesmar FIP or non-Artesmar, which works for the two municipalities of Sablayan and Mamburao. The sample selection equation is represented by:

$$z^{*}=\alpha^{’}\text{w }+\text{ u}$$

$$z=1\left[z^{*}>0\right]$$

The variable z* is a shadow variable ruling the fisher participation in Artesmar FIP, *α′* is a coefficient of the selection process, *w* is the explanatory variables known to influence the selection decision, and μ is the random error term of sample selection equation that is normally distributed. When *z* = 1, the fisher belongs to the category ‘non-Artesmar’ (and z = 0 for the Artesmar FIP) since further analysis will be done for a group of fishers not involved in the Artesmar FIP. All variables in Table 1 are used in the selection equation.

Next is to use the result in the first equation to calculate the inverse Mills ratio, a selection hazard that is added as an explanatory variable in the second equation to remove the sample selection bias. Similar to Heckman’s two-stage sample selection model and to other studies (e.g. [59–63]) that employed the same methodology, the second stage is an ordered probit equation. Since the second stage applies for the six regions where PPTST FIP operates (excluding Artesmar FIP in the analysis), individual fishers in Mamburao and Sablayan have values in their inverse Mills ratio while fishers in the remaining four municipalities have inverse mills ratio set to zero. The ordered probit equation is:

$$y^{*}=\beta^{\prime }x+\gamma \cdot \lambda +\varepsilon ,$$

$$y=j\;if\;\mu_{i-j}<y^{*}\leq \mu_{j}.$$

The y* represents a shadow variable ruling the ordered stages of participation to which non-Artesmar fishers belong: *β’* represents the coefficient of outcome explanatory variables *x*, *γ* is the selectivity bias, *λ* is the inverse Mills ratio, and ε is the random error term for the ordered probit equation. The y = j corresponds to the exact stage of participation in non-Artesmar, and is decomposed as follows: 1(y = 1) for non-participation, 2 for partial participation in the PPTST FIP, and 3 for full participation in the PPTST FIP. The variables in Table 1 are used in the ordered probit equation.

Before running the probit and the ordered probit models, two steps were conducted. First, all independent variables were checked for multicollinearity using the Variance Inflation Factor (VIF), which in this case gave values well below 10 for all explanatory variables (see S2 Table under Supporting Information). This means that all explanatory variables exhibit low multicollinearity so all variables are retained in the model. Second, the heteroskedasticity of the model was tested using the Breusch-Pagan test to determine whether the variance of the error terms increases or decreases with explanatory variables. The heteroskedasticity test shows a significant result (see S3 Table), implying that running the model using robust standard errors reduces the variance of the error terms.

Finally we added two extra steps to the model to test the robustness of our result, as shown in the supplementary information (see S5 and S6 Tables). First, we checked for the significance of the inverse Mills ratio using the samples in Mamburao and Sablayan. Second, we ran a separate ordered probit model in the second stage that does not account for inverse Mills ratio for comparison with the one that does account for inverse Mills ratio.

## Results

First the modelling results will be presented for the first stage decision on participation in the Artesmar FIP, followed by the results for the second stage decision on participation in the PPTST FIP and the ordered outcome of non-participation in FIP, partial and full-participation in PPTST FIP.

### Participation in Artesmar FIP

Table 4 shows all independent variables that might explain participation of fishers in the Artesmar FIP, together with the corresponding coefficients, standard error, and Z-value. The coefficients are interpreted based on the direction of the effect of the variables [56]. For instance, a positive coefficient means that a variable increases the participation of fishers in Artesmar FIP while a negative coefficient reduces the participation of fishers in Artesmar FIP. It is not possible to use the value of these coefficients in estimating the increase or decrease of participation because the coefficients are not derived using a linear model. The inverse Mills ratio shows insignificant result which means that there is no evidence that selection bias is quantitatively important.

**Table 4:** Results of the probit model in the first stage using data from Sablayan and Mamburao fishers.

	Coefficients	Standard Error	Z
FIP^a^			
*Individual personal capabilities*			
Fishing years	0.005	0.014	-0.35
Education	0.156	0.303	-0.51
*Individual firm capabilities*			
Initial investment	0.089***	0.029	-3.082
Boat ownership	-0.391	0.351	1.11
Boat capacity	0.035	0.085	-0.42
Fishing trips	0.011	0.044	-0.25
Type of fishing employment	-0.836***	0.300	2.78
Operating distance	-0.003	0.005	0.53
Fishing days	0.070	0.053	-1.32
*Collective capabilities*			
Membership to Association	-0.269	0.330	0.82
Financing operation	-0.276	0.279	0.99
Trainings and subsidies	-0.549	0.414	1.33
*Individual risk attitude and socio demographic*			
Risk attitude	1.132***	0.293	-3.86
Age	0.017	0.018	-0.98
Number of family members	0.259***	0.069	-3.78
Other sources of income	0.119	0.279	-0.42
constant	3.80 ***	0.941	4.03

*N* = 220; Wald chi(16) = 47.60; prob>(chi)2 = 0.000; inverse mills ratio = 0.16

* Statistical significance at α = 0.10

** Statistical significance at α = 0.05

*** Statistical significance at α = 0.01

^a^For reading the result, let 1 = Artesmar FIP and 0 = non-Artesmar

Note: The fisher data involve municipalities of Sablayan and Mamburao. The actual number of surveys is 222, but Stata eliminates observation with incomplete responses.

The results presented in Table 4 show that some explanatory variables have significant effects on fishers’ choice for Artesmar FIP over non-Artesmar. The fit of the model based on the probit equation is P > χ^2^ = 0.00, which implies a good model fit at α = 0.01. Consistent with our hypothesis, the initial investment for fishing boats, risk attitude, and number of family members are significant and positively contribute to the fishers’ choice to participate in the Artesmar FIP. Increasing initial investment for fishing boat reflects higher capabilities of fishers in terms of boat size, boat engine, and fishing equipment. These capabilities enable fishers to deliver tuna in the chain, therefore increasing their likelihood of choosing the Artesmar FIP and their chances of gaining higher income to recover their investments. A higher value for risk attitude of fishers as manifested by fishers preference to sell through quality buying, increases the likelihood of fishers to participate in the Artesmar FIP. A higher number of family members indicates that fishers also have an increase likelihood to participate in the Artesmar FIP. Based on field observations and interviews, fishers participating in Artesmar FIP often operate as part of a large scale family business. As shown in the literature [64], family members in the fishing business reduce fishers’ risks by having trusted employees, by developing the capabilities of the family members in order to succeed the business in the future, and by having confidence that a fisher contributes to the welfare of the family member.

Contrary to what was expected, the type of fishing employment has a negative effect on participation in the Artesmar FIP. The results show that the more fishers operate part-time or target species aside from tuna, the more likely it is that they will participate in the Artesmar FIP. Most part-time tuna fishers shift from tuna fishing to catching other species during the low season for tuna catches in order to recover their costs of operation. Moreover, based on field interviews, some fishers believe that tuna stocks are declining over time, and therefore search for alternative catch to balance the risks of going out to sea.

### Participation in PPTST FIP

The results of the ordered probit model on the degree of participation of fishers in the PPTST FIP are presented in Table 5. Variables such as type of fishing employment, operating distance, membership to an association, and risk attitude have positive and significant effects on the degree of fishers’ participation. The type of fishing employment increases the likelihood of full-participation in PPTST FIP when fishers target tuna on a full-time basis. Fishers specializing in tuna may have better skills in catching and improving tuna as compared to diversified fishers, thus enabling them to comply with the product requirements of PPTST FIP and therefore increasing their chance of generating higher income. As the operating distance increases, the likelihood of full participation in the PPTST FIP also increases. Fishers also note that the declining catch in municipal water (<15 km from seashore) drives them further seaward to search for better fishing grounds. Doing so increases their chances of catching and delivering tuna in the PPTST FIP, which in turn increase their chances of gaining higher income.

**Table 5:** Results of the estimation of the ordered probit model in six municipalities of Occidental Mindoro.

	Coefficients	Standard Error	Z
Stages^a^			
*Individual personal capabilities*			
Fishing years	0.012	0.007	1.52
Education	0.089	0.152	0.58
*Individual firm capabilities*			
Initial investment	0.074	0.061	1.21
Boat ownership	0.227	0.208	1.09
Boat capacity	-0.037	0.071	-0.53
Fishing trips	-0.009	0.014	-0.65
Type of fishing employment	0.407**	0.188	2.16
Operating distance	0.005**	0.003	2.03
Fishing days	0.026	0.040	0.65
*Collective capabilities*			
Membership to Association	1.484***	0.185	8.01
Financing operation	-0.241	0.159	-1.52
Trainings and subsidies	0.078	0.196	0.4
*Individual risk attitude and socio demographic*			
Risk attitude	0.770***	0.254	3.03
Age	0.003	0.008	0.41
Number of family members	-0.034	0.038	-0.88
Other sources of income	-0.304*	0.157	-1.93
Inversemills1	-1.083	0.771	-1.4
Cut1	1.04	0.455	
Cut2	2.57	0.474	

*N* = 296; Log likelihood = -223.4; LR chi(17) = 168.5 P > χ^2^ = 0.000***

* Statistical significance at α = 0.10

** Statistical significance at α = 0.05

*** Statistical significance at α = 0.01

^a^ The fisher data involves the six PPTST FIP municipalities, excluding the fishers in Artesmar FIP

The membership to a fisher association also increases the likelihood of fishers to move from non-participation to full-participation in PPTST FIP. Based on interviews, fishers in the PPTST FIP perceive that joining a fisher association will help them improve the quality of their tuna, and will finally lead to higher tuna prices. The fisher association increases the perceived benefits of fishers since the association helps fishers to generate funding and subsidy such as fishing aids from the government, which improves their fishing activities. Moreover, a fisher association also organises activities such as the marine guards to prevent illegal fishing and conservation activities as part of the improvement process in the FIP. Finally, the risk attitude of fishers shows a positive effect in terms of pushing fishers to fully participate in the PPTST FIP. This means that fishers are willing to sell more through quality buying because these fishers are confident that they acquired the knowledge and skills to improve the quality of tuna catches, which in turn increases the probability of getting higher prices for the fish they land.

Having alternative sources of income has a negative and significant effect on the participation of fishers in the PPTST FIP as compared to those fishers who solely rely on fishing as a source of income. Based on interviews, and consistent with literature related to fishing as a form of employment (e.g [49]), some fishers consider fishing as the poorest among other forms of employment and would like to move away from fishing if possible. Therefore, having other sources of income such as farming and small enterprises leads them to prioritize alternative sources of income rather than improving their fishing practices to satisfy FIP requirements.

**Fig 3:**
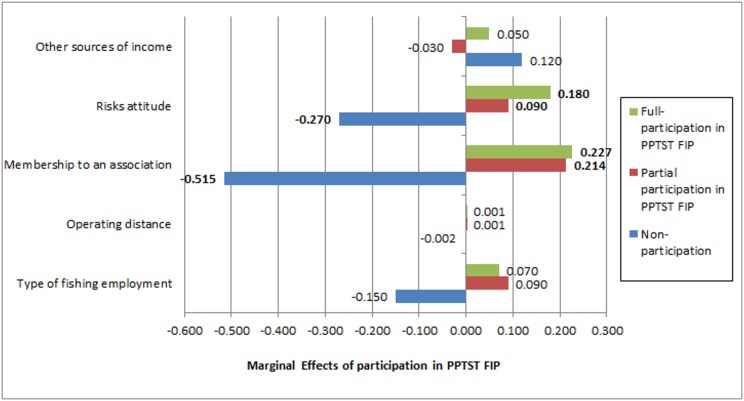
The marginal effects of significant variables in second stage decision after ordered probit model. Note: The numbers in bold represent the marginal effects significant at α = 0.05.

Finally, the significant variables in the ordered probit model are further analysed by deriving their marginal effects, which show the change in probability when an independent variable increases by one unit. Calculating marginal effects in the second stage decision shows both the patterns and requirements for fishers to move from one participation stage to another. Fig 3 shows these marginal effects of the type of fishing employment, operating distance membership to an association, risk attitude, and other sources of income based on non-, partial-, and full-participation in the PPTST FIP (also refer to S3 Table for detailed information). The vertical axis of the graph represents the values of marginal effects from low to high, while the data labels or numbers in bold represent the significance of marginal effects at α = 0.05. Taking the membership to a fisher association as an example, the graph shows that when any fisher is a member of a fisher association, the probability of not participating reduces by 51.5%, while the probability of partial participation increases by 21.4%, and of full participation increases by 22.7%. In another example, an addition of one km to the 35.5 km average operational distance (see Table 3) will reduce the non-participation of fishers by 0.2% (see Fig 3).

Aside from the direct interpretation of the marginal effects, the graph also shows the trends of marginal effects in selecting for partial or full participation in the PPTST FIP. The five variables show an increasing trend, which means that they generally enhance the participation of fishers from non- to full-participation in the PPTST FIP. The variables membership to a fisher association and risk attitude are highly significant in non, partial, and full-participation in PPTST FIP at α = 0.01. This means that both variables are highly important in improving the participation of fishers in the PPTST FIP. The type of fishing employment, operating distance, and financing operation are significant at α = 0.05 and at 0.10. It is also notable that among the five variables, the membership to a fisher association shows the highest marginal values, making it highly important for both partial and full participation. The majority of the partial participants are able to get funding from the government to improve their livelihoods through a fisher association. Full participants also benefit by being members of a fisher association by having regulatory support to fight IUU fishing in their area, thus ensuring greater legality of fish caught and traded.

## Discussion

Based on these results we now further elaborate on how the capabilities of fishers influences fisher decision making for different levels of participation in top-down and bottom-up comprehensive FIPs, and on the influence these factors have on the design of different FIPs as a means of reaching goals of improved fishery practices and fisheries management.

### Capabilities, risk and FIP participation

The results show that different types of capabilities influence the decision of fishers to join different kinds of FIPs. First, individual firm capabilities, such as initial investment and type of fishing employment, increase the likelihood for fisher participation in response to market-oriented FIPs, which provide economic incentives through dock side transactions based on quality. These individual firm capabilities are more likely to be exhibited by those with considerable financial capacity, owning and financing several fishing boats. Moreover, these fishers are also more likely to be engaged in other chain-related activities. However, consistent with now wider observations of how economic decisions are embedded in wider social or community relations [65, 66], the results also show that these individual firm capabilities are not independent from wider social relations in which these firm actors find themselves in fishing communities. For example, socio-demographic variables, such as the higher number of family members, are also shown to play a role in the probability of participating in market-based FIPs possibly because familial ties reduce the risk associated with individual choices to invest in complying with FIP requirements [64, 67].

Second, collective capabilities, such as membership to an association, are more likely to increase participation in the bottom-up PPTST FIP. The results also show that full participation in development-oriented FIPs remains dependent on the characteristics of the individual fishing firm. The results indicate that fishers fully participating in PPTST FIP have a greater degree of operation, for instance by their large capacity to finance high costs of operation at a farther distance and through their capability to cope with risks associated with full-time tuna fishing particularly from March to September where catches are lower (see for example [68]). However, partial participation is dominated by those fishers with smaller ‘one-man, one-boat’ operations, characterised by fishing at shorter distance from shore, and who are dependent on collective capabilities supported by fishing associations and *Casas* (see [69] for comparable results). The consequence appears to be that despite the bottom-up oriented nature of the PPTST FIP, targeting a wider group of smaller scale and relatively poorer fishers, full participation appears to remain linked to those with higher individual firm level capabilities. This supports the idea of “elite capture” of benefits [70], meaning that fishers with a high level of individual capabilities, in general, benefit from participation in FIPs.

In addition to capabilities, the results indicate that risk attitude also influences the decision of fishers to participate in the two FIPs. First, the results indicate that less risk averse fishers are more likely to participate in the top-down Artesmar FIP. Building on work around fisher behaviour in response to economic risk [39, 40, 46], this finding indicates that those fishers choosing for Artesmar FIP do so in order to not only generate higher income, but more importantly to reduce the lack of transparency and high price volatility inherent in the landings sites controlled by *Casas*. This means that the high price in Artesmar FIP reduces the risks of fishers against lower fish prices, as long as these fishers satisfy the fish quality requirement. In contrast, those choosing for the bottom-up PPTST FIP were not able to reduce this risk, as seen in many small-scale developing country fisheries [71, 72], because most buyers involved were not moving to quality buying and maintained many of the same debt-tied relations.

### Consequence for FIP design

Our results hold consequences for the design of FIPs, as well as the relationship between different FIP models. First, reflecting wider discussions on market-based approaches to environmental improvement (such as eco-certification, see [73]), top down comprehensive FIPs requiring high level of individual capabilities have a higher risk of being selective of those fishers who can more easily comply with sustainability standards in exchange for immediate market access [6]—with the consequence of reducing the potential for overall fishery improvement. In contrast, bottom up comprehensive FIPs fostering collective capabilities appear to be more inclusive of a larger group of fishers, but with the potential consequence of delivering lower overall improvements to the fishery as a whole. Balancing these trade-offs is at the core of the differentiation on how different FIP models are evolving [19].

Second, the results indicate that the design of FIPs also influences the delivery of incentives to fishers, which also affects inclusiveness and overall impact. As argued above, top-down FIPs appear to be better able to deliver more transparent price signals to fishers. The case of the Artesmar FIP shows that companies like Meliomar invest in vertical coordination and in doing so control the chain from production to export. They are thus better able to set higher prices for higher quality fish and to transmit this price incentive to fishers [74, 75]. On the other hand, bottom-up FIPs with weaker relationships between retailers and fishers may result in weaker control over chain coordination. In addition, the on-ground implementation by actors operating outside the chain such as WWF, appears to result in weaker delivery of price-related incentives. Based on field interviews and observation, WWF serves as a facilitator outside the chain that links fishers to other seafood stakeholders. However, WWF does not have influence over the price of fish. Nevertheless, supporting other observations [6, 14], bottom-up comprehensive FIPs do appear to offer more durable support from government given their closer relationship during implementation.

Finally, these results open up questions around the degree of complementarity and competition that might exist between FIP models in relation to inclusion and improvement. While the results point to the differences between top-down or bottom-up comprehensive FIPs, it may not necessarily be the case that these or other FIP models are opposed or in competition. Instead, building on nascent ideas of FIP design [19], two FIP models operating in one place points to a range of potential complementarities in terms of delivering incentives to fishers and improving the governance of the fishery. Here the more stringent top-down market-oriented approach could target fishers with individual firm capabilities, while bottom up ‘development-oriented’ FIPs could provide a more inclusive approach. By targeting different capabilities, and setting different incentives, the two models could work in tandem to provide overall greater gains in fishery improvement.

## Conclusion

Our results indicate the overall importance of considering fisher capabilities in understanding decisions of fishers to participate in different kinds of FIPs. The individual firm capabilities stimulate fishers to participate in strongly market oriented top-down FIPs while collective capabilities enable fishers to improve their practices and to participate in bottom-up FIPs aimed at wider fishery and community development goals. These types of capabilities and FIP models have consequences for the inclusiveness of different fishers, for which three insights are generated in this study. First, the top-down FIPs appear to be more exclusive, given that there are a few numbers of fishers who have the capabilities and lower risk aversion to comply with its requirements. Second, bottom-up FIPs appear to be more inclusive, aiming to provide broad support to the development of fisher capabilities and the institutional support provided to them to improve their practices on the water. Third, the two FIP models exhibit competing inclusion when implemented in the same fishery, yet also exhibit potential complementarity in terms of delivering incentives and improving governance of the fishery.

We also conclude that in order to increase the participation of fishers, those designing FIPs need to not only recognize the economic utility that a fisher could derive from participation, but also to identify and build fishers capabilities to participate in a FIP. Moreover, FIPs must also balance both the deliverance of incentives to individual fishers and the improvement in the governance of the fishery in order to enhance participation of fishers and thus achieve high sustainability impact. Future research may elaborate on the implementation of FIPs to poor and well performing fishers and to different fisheries (e.g. tuna or other species), and on their consequences to the inclusiveness and fishery improvement. Finally, focusing on ways to enhance the participation of fishers in FIPs and in other private incentive mechanisms is also relevant to achieve higher sustainability outcomes.

## Supporting Information

S1 File Estimation of the sample size. (PDF)

S2 File Survey forms on household fishers. (PDF)

S1 Table Data of individual fisher surveys in Occidental Mindoro fishery. (XLSX)

S2 Table Testing the explanatory variables for multi-collinearity using a Variance Inflation Factor (VIF). (PDF)

S3 Table Testing the explanatory variables for heteroskedasticity. (PDF)

S4 Table Summary of the marginal effects of ordered probit model with sample selection in second stage decision making. (PDF)

S5 Table Ordered probit model of fisher data from Sablayan and Mamburao, Occidental Mindoro with inverse Mills ratio. (PDF)

S6 Table Ordered probit model of fisher data from Occidental Mindoro without inverse Mills ratio. (PDF)
