# Supplementary material for: Fisher-Level Decision Making to Participate in Fisheries Improvement Projects (FIPs) for Yellowfin Tuna in the Philippines
Source: PLoS One. 2016 Oct 12;11(10):e0163537. doi: 10.1371/journal.pone.0163537 (PMC5061383; doi:10.1371/journal.pone.0163537)
Supplement: S1 File — (PDF) [file pone.0163537.s001.pdf]

S1 File. Estimation of the sample size

The sample size was calculated starting from unknown population equation:

$$ss = (Z\text{-}score)^2 * sd * (1 - sd) / (c)^2 \qquad (1)$$

where *Z-score* is the standard score in statistics, *sd* is the standard deviation, expressed as decimal, and *c* is the confidence interval, also expressed as decimal. We use the 95% confidence level with *Z-score* of 1.96 and a confidence interval of ± 5%. The recommended standard deviation of 0.5 is used before the survey is administered as it ensures that the samples will be large enough (REF). The recommended sample size for unknown population is 384. Since the population of fishers in the area is known, the sample size is adjusted based on unknown population equation (Field, 2003):

$$new\ ss = ss/(1 + ss - 1/population) \qquad (2)$$

In equation 2, the *new ss* represents new sample size while *ss* is the required sample size in equation 1. The adjusted sample size of for known population is 350 samples.
