# Supplementary material for: Fisher-Level Decision Making to Participate in Fisheries Improvement Projects (FIPs) for Yellowfin Tuna in the Philippines
Source: PLoS One. 2016 Oct 12;11(10):e0163537. doi: 10.1371/journal.pone.0163537 (PMC5061383; doi:10.1371/journal.pone.0163537)
Supplement: S2 File — (PDF) [file pone.0163537.s002.pdf]

SURVEY FORM FOR SHORT- AND LONG-TERM DECISION MAKING OF HOUSEHOLD/INDIVIDUAL FISHERS

Dear Madame/Sir:

Good day!

I am Frazen G. Tolentino, Phd student at Wageningen University (The Netherlands). I am currently conducting research on the effects of incentive mechanisms such as MSC certifications, Fishery Improvement Projects or FIPs, and private branding strategy of ISSF member brands, to short- and long-term decision making of fishers. The goal of my research is to provide insights on which decisions, such as how much to catch, how much resources to use, and invest or disinvest in fishing technologies and fleets, will help fishers reach their objectives. In line with this, I would like to request for your participation in a survey. The survey may take an hour of your time. Please rest assured the information you provided will be used for academic and research purposes only. At the end of the survey, I'll kindly ask you to sign the form as proof of your participation in the survey and that you agree to use the information for academic and research purposes.

Thank you very much.

P.S.: In case of further information or clarification, provided is my contact information:

Frazen Tolentino
+63927-248-1512
Frazen.tolentino@wur.nl, frazentolentino@gmail.com

Business Economics Group
Wageningen University, The Netherlands

Name of interviewer: **Date and time of interview:**
Signature of fisher:

A. HOUSEHOLD FISHERS' CHARACTERISTICS

A.1. SOCIO-DEMOGRAPHIC ASPECT

Name:			
Address:			
Contact Information (cell no.):			
Gender (mark X):	O=Male O=Female	Age:	
Number of years in fishing:			
Number of family members:			
Other sources of income (mark X and specify associated monthly income):	O=fishing other species , ________________________<br>O= farming, ___________________________________<br>O= driving tricycle, ____________________________<br>O=grocery stores/small business, __________________<br>O=construction works, ___________________________<br>O=others, please specify __________________________		
Highest educational attainment (mark X, specify level and degree, if applicable)	O=no formal education O=primary (elementary)<br>O=secondary (high school) O=vocational degree<br>O= college degree (BS or BA) O=others, specify		

B. MATERIAL RESOURCES

B.1. FISHING BOATS

No. of fishing boats	
Name of fishing boats/vessels	
Initial investment (PhP)	
Replacement value (PhP)	
Boat repair (PhP/year)	
Age of the boat (year)	
Number of boat crew (including captains)	
Fishing licenses or registration (PhP/year)	
Vessel weight (metric tons)	
Length size (m)	
Fish capacity (in kg)	
Number of compartments/bins	

B.2. VESSEL OWNERSHIP (mark X)

O=owner	O=boat captain	O=passenger
If owner, how did you finance your vessels?		
O=personal savings	O=bank loans	O=fisher's cooperative
O=through relatives	O=others, specify______________	

Amount loan: ________________________________
Interest Rate: ________________________________
Monthly Payment: ____________________________

B.3. FISHING GEARS (What fishing gears do you use? Mark X)

Fishing gears	Costs:
O= handline	
O=hook-and-line	
O=jigger	
O=pole and line	
O=others, specify ____________________	
Length size: __________________________	
Number of traps: ____________________	

B.4. FISHING TECHNOLOGIES (mark X and determine the amount of investments, life span, number of years used, maintenance costs)

	**Investment (PhP)**	**Life span**	**No. Of years used**	**Maintenance costs**
O= sonar radar				
O=compass				
O=GPS tracking device				
O=radio				
O=generator				
O=others, specify				

C. FIP REQUIREMENTS

A. Fisherfolk and Vessel registration
A.1 Are you a registered fisherfolk in your respective municipality? O=No O=Yes
If not, reason why? ____
A.2 Vessel registration (in Table B1)

B. Traceability
B.1 Do you use catch documents O=No O=Yes
If yes, what are the information provided in catch documents? ________________________
____
B.2 Do you use tuna tags? O=No O=Yes
If yes, what are the information provided in tuna tags? ______________________________
____

C. Fish Quality

C.1. What's your mode of selling tuna? O=Straight buying O=Quality Buying

Price in Straight Buying (PhP) ________________________

Price in Quality Buying: Good quality: ______________ Reject: _____________________

D. Trainings

Are you aware of FIP	O=No O=Yes
If yes, which FIP are you aware of?	O=PPTST O=Artesmar
When did you join FIP?	
Reasons for joining?	
Are you a member of fishers' cooperative/association? (mark X)	O=No O=Yes
If yes, name of association	
Position in association	
Activities of association (mark X):	O=provide credit
	O=provide trainings
	O=provide marketing services
	O=others, specify

C.2. Trainings and Education (please shade and identify the name of trainings)

O Capacity buildings, ____

O = Marketing and value-adding activities on tuna, ________________

O=New fishing technology, ___________________________________

O=Seminars on fishery management, ___________________________

C.3. Subsidies from the government (mark X those that are received)

Subsidies from the government	O= fuel subsidy
	O=new fishing gears
	O=new boat motors
	O=others, please specify, ____________________________

SHORT-RUN OBJECTIVES (Maximise fishing income, minimise risks)

A. FISHING TRIP INFORMATION

Name of fishing Ground	
Fishing trip (in hours or days, one way)	
Number of fishing days	
Number of fishing trips in a month	
Name of unloading port	
Distance of fishing ground to unloading port	

B. COSTS per FISHING TRIP

	Usage	Costs
Fuel consumption for boat's motor (L)		
Gasoline consumption for generator (L)		
Ice (Blocks)		
Baits (bottles)		
Workers expenses (include foods, cigarettes, water, medicines)		
Total Fishing Costs (PhP)		

Worker's wage: If sharing system what are the percentages for:

boat owners ________ captains _________ crews _________
how much do you normally get per fishing trip? _____________

How do you finance your fishing operation? O=Own financing O=Casas

E. FISHING OUTPUTS

Catches (2013 and 2014):	Yellowfin		Skipjack		Small pelagics		for home consumption (Kgs)
Highest catch							
Average catch							
Lowest catch							

Tuna Prices in PhP (2013 and 2014):	Yellowfin		Skipjack		Small pelagics		for home consumption (Kgs)
Highest price							
Average price							
Lowest price							

Who are the buyers of tuna? (mark X)	O=Casas
	O= Local consumer
	O=Restaurants
	O=others, specify ______________________________

Break-even catch (in kg) per fishing trip ____
Difference in price before and after joining FIP? _______________________________________
Difference in fish volume before and after joining FIP? _________________________________

H. ALTERNATIVE ACTIVITIES:
During low season of tuna, what species do you catch? ___________________________________
Where do you fish? ____

What will make you exit tuna fishery? (Rank from 1-5)	
Low supply	
Stiff competition	
Stringent regulation	
Age	
Others	

Alternative activity when you exit tuna fishery? (Mark X)

O=Farming	O=Grocery store	O=tricycle driving	O=other business venture	O=construction work	O=others, specify _____________________

-- Thank You -------------------------------------
