# Supplementary material for: Fisher-Level Decision Making to Participate in Fisheries Improvement Projects (FIPs) for Yellowfin Tuna in the Philippines
Source: PLoS One. 2016 Oct 12;11(10):e0163537. doi: 10.1371/journal.pone.0163537 (PMC5061383; doi:10.1371/journal.pone.0163537)
Supplement: S2 Table — (PDF) [file pone.0163537.s004.pdf]

S2 Table. Testing the explanatory variables for multi-collinearity using a Variance Inflation Factor (VIF)

. vif

Variable	VIF	1/VIF
-------------+----------------------		
Fishing years	1.65	0.606082
Education	1.09	0.920212
Membership to association	1.45	0.691689
Training	1.54	0.650978
Initial investment	1.34	0.74811
Boat ownership	1.52	0.657019
Boat capacity	1.38	0.725184
Financing operation	1.19	0.842799
Fishing trips	2.24	0.446546
Fishing employment	1.45	0.690904
Operating distance	1.29	0.777075
Fishing days	2.18	0.459655
Risk attitude	1.19	0.840245
Age	1.76	0.568175
Family members	1.14	0.879963
Other sources of income	1.1	0.909165
-------------+---------------------		
Mean VIF		1.47
