# Supplementary material for: Fisher-Level Decision Making to Participate in Fisheries Improvement Projects (FIPs) for Yellowfin Tuna in the Philippines
Source: PLoS One. 2016 Oct 12;11(10):e0163537. doi: 10.1371/journal.pone.0163537 (PMC5061383; doi:10.1371/journal.pone.0163537)
Supplement: S3 Table — (PDF) [file pone.0163537.s005.pdf]

S3 Table. Testing the explanatory variables for heteroskedasticity

```
. hettest

Breusch-Pagan / Cook-Weisberg test for heteroskedasticity
         Ho: Constant variance
         Variables: fitted values of stages

         chi2(1)      =     1.90
         Prob > chi2  =   0.1679
```
