# Supplementary material for: Fisher-Level Decision Making to Participate in Fisheries Improvement Projects (FIPs) for Yellowfin Tuna in the Philippines
Source: PLoS One. 2016 Oct 12;11(10):e0163537. doi: 10.1371/journal.pone.0163537 (PMC5061383; doi:10.1371/journal.pone.0163537)
Supplement: S4 Table — (PDF) [file pone.0163537.s006.pdf]

S4 Table. Summary of the marginal effects of ordered probit model in second stage decision making

Variable	dy/dx (part=1)	Std. Err.	z	dy/dx (part=2)	Std. Err.	z	dy/dx (part=3)	Std. Err.	z
	0.4125			0.4924			0.0951		
Individual personal capabilities									
Fishing years	-0.0046	0.0031	-1.52	0.0027	0.0018	1.48	0.0023	0.0013	1.83
Education	-0.0345	0.0590	-0.58	0.0195	0.0333	0.58	0.0240	0.0234	1.03
Individual firm-capabilities									
Initial investment	-0.0286	0.0237	-1.21	0.0162	0.0136	1.19	0.0124	0.0104	1.19
Boat ownership	-0.0889	0.0816	-1.09	0.0520	0.0499	1.04	0.0368	0.0325	1.13
Boat capacity	0.0145	0.0276	0.53	-0.0082	0.0156	-0.53	-0.0063	0.012	-0.53
Fishing trips	0.0036	0.0055	0.65	-0.0020	0.0031	-0.64	-0.0015	0.0024	-0.65
Type of fishing employment	-0.1564**	0.0711	-2.2	0.0852**	0.0390	2.18	0.0712**	0.0351	2.03
Operating distance	-0.0020**	0.0010	-2.02	0.0011*	0.0006	1.94	0.0008**	0.0004	1.98
Fishing days	-0. 0102	0.0157	-0.65	0.0058	0.0089	0.65	0.0125	0.0048	2.63
Collective capabilities									
Membership to association	-0.5155***	0.0525	-9.81	0.2136***	0.0402	5.32	0.2272***	0.0414	5.49
Financing operation	0.0937	0.0613	1.53	-0.0528	0.0351	-1.77	-0.0426	0.0241	-1.77
Trainings and subsidies	-0.0301	0.0758	-0.4	-0.0167	0.0415	0.4	-0.0146	0.0288	-0.51
Individual perception of risks and socio demographic									
Risk attitude	-0.2661***	0.0737	-3.61	0.086***	0.0240	3.6	0.1797***	0.0767	2.34
Age	-0.0013	0.0033	-0.41	0.0008	0.0019	0.41	0.0006	0.0014	0.41
Family members	0.0130	0.0149	0.88	-0.0074	0.0085	-0.87	-0.0057	0.0065	-0.87
Other sources of income	0.1183*	0.0612	1.93	-0.0338*	0.0307	-1.1	-0.0502*	0.0261	-1.92
Inverse mills ratio	0.4216	0.3003	1.4	-0.2384	0.1731	-1.38	-0.1832	0.1323	-1.38
Cut 1	.4530								
Cut 2	.0995								

N=316; Log likelihood = -304.17358; LR chi2(12)=156.04; Prob > chi2=0.0000

*, **, *** significance at α=0.10, 0.05, and 0.01 respectively

The part=1, =2, and =3 represent non-participation, partial-, and full participation in PPTST FIP respectively

The following Cut corresponds to: 1=non-participation, 2=partial participation, 3=full participation
