# Supplementary material for: Fisher-Level Decision Making to Participate in Fisheries Improvement Projects (FIPs) for Yellowfin Tuna in the Philippines
Source: PLoS One. 2016 Oct 12;11(10):e0163537. doi: 10.1371/journal.pone.0163537 (PMC5061383; doi:10.1371/journal.pone.0163537)
Supplement: S5 Table — (PDF) [file pone.0163537.s007.pdf]

S5 Table. Ordered probit model of fisher data from Sablayan and Mamburao, Occidental Mindoro, with inverse Mills ratio

Ordered probit regression		Number of obs = 200	
		LR chi2(16) = 105.48	
		Prob.chi2 =0.000	
Log likelihood = -138.76397		Pseudo R2 = 0.3088	
Stages	Coefficients	Standard error	Z
Fishing years	0.016	0.011	1.46
Education	-0.036	0.209	-0.17
Membership to association	1.68	0.252	6.65***
Training	0.148	0.259	0.57
Initial investment	0.036	0.072	0.5
Boat ownership	0.485	0.246	1.97**
Boat capacity	-0.052	0.084	-0.62
Financing operation	-0.296	0.200	-1.48
Fishing trips	-0.037	0.024	-1.55
Fishing employment	0.255	0.236	1.08
Operating distance	0.004	0.003	1.52
Fishing days	0.051	0.053	0.96
Risk attitude	0.957	0.410	2.33**
Age	-0.003	0.010	-0.28
Family members	-0.007	0.065	-0.11
Other sources of income	-0.422	0.204	-2.07**
Inverse Mills Ratio[1]	-0.865	1.1018	-0.79
/cut1	1.20	0.573	
/cut2	2.31	0.606	

[1]The inverse Mills ratio is insignificant in ordered probit model of fisher data from Sablayan and Mamburao, Occidental Mindoro
