# Supplementary material for: Fisher-Level Decision Making to Participate in Fisheries Improvement Projects (FIPs) for Yellowfin Tuna in the Philippines
Source: PLoS One. 2016 Oct 12;11(10):e0163537. doi: 10.1371/journal.pone.0163537 (PMC5061383; doi:10.1371/journal.pone.0163537)
Supplement: S6 Table — (PDF) [file pone.0163537.s008.pdf]

S6 Table. Ordered probit model of fisher data in Occidental Mindoro without inverse Mills ratio

Ordered probit regression		Number of obs =	296
		LR chi2(16) =	166.46
Log likelihood = -224.41025		Pseudo R2 =	0.2705
Stages	Coefficients	Standard error	Z
Fishing years	0.010	0.008	1.3
Education	0.067	0.151	0.45
Membership to association	1.54	0.181	8.54***
Training	0.147	0.189	0.78
Initial investment	0.052	0.058	0.89
Boat ownership	0.292	0.203	1.44
Boat capacity	-0.047	0.070	-0.68
Financing operation	-0.241	0.158	-1.52
Fishing trips	-0.011	0.014	-1.82
Fishing employment	0.490	0.179	2.73***
Operating distance	0.006	0.002	2.31**
Fishing days	0.007	0.038	0.17
Risk attitude	0.597	0.221	2.7***
Age	0.002	0.008	0.28
Family members	-0.054	0.035	-1.52
Other sources of income	-0.313	0.157	-2**
/cut1	.913	.446	
/cut2	2.44	.464	
